# Precision versus pitfalls: Navigating margin management in radiotherapy amidst technological advancements

**DOI:** 10.1002/acm2.14465

**Published:** 2024-07-22

**Authors:** Savino Cilla, Erika Galietta, Costanza M. Donati, AFM Kamal Uddin, Alessio G. Morganti

**Affiliations:** ^1^ Medical Physics Unit, Gemelli Molise Hospital‐Università Cattolica del Sacro Cuore Campobasso Italy; ^2^ Department of Medical and Surgical Sciences (DIMEC) Alma Mater Studiorum University of Bologna Bologna Italy; ^3^ Department of Radiation Oncology National Institute of ENT Dhaka Bangladesh; ^4^ Radiation Oncology IRCCS Azienda Ospedaliero‐Universitaria di Bologna Bologna Italy

Dear Editor,

In the evolving landscape of radiotherapy (RT), the pursuit of precision has led to significant advancements, from 2D to sophisticated modalities like IMRT, VMAT, and SBRT. These innovations have undeniably improved targeting accuracy, reducing treatment margins and minimizing toxicity. However, this progress brings to light a critical concern: the risk of geographic misses due to overly reduced margins, particularly in the absence of adequate image‐guided radiotherapy (IGRT) systems.

Our experiences, particularly during a recent educational initiative on prostate cancer in a low‐intermediate income country, revealed a prevailing belief among some colleagues that advancements in RT inherently allow for the use of reduced irradiation margins, to define smaller target volumes and thus allow dose escalation with reduced toxicity. This perception underscores a widespread optimism towards the precision of modern RT techniques but may overshadow the intricate balance required between minimizing toxicity and ensuring adequate tumor coverage.

Evidence from various studies across different cancer types (Table 1), including prostate^1^, ^2^, ^3^ and head and neck cancers,^4^, ^5^ suggests that reduced margins, despite the precision of modern techniques, may lead to suboptimal treatment outcomes. Notably, studies have shown that smaller treatment margins can be linked to worse outcomes in certain cases. For example, the efficacy and toxicity of conformal RT in treating orbital lymphoma were explored, revealing that partial orbit RT, aiming for reduced toxicity, was associated with a significant occurrence of intra‐orbital recurrence compared to whole orbit RT.^6^

**TABLE 1:** Summary of the cited studies.

Author/year	Setting	Comparison	Results
Pfeffer et al., 2004^6^	Orbital lymphoma	2D versus 3D	LR: 0% versus 33%
Engels et al., 2009^3^	Prostate	CTV‐PTV: 6−10 versus 3−5 mm (fiducials)	5y bRFS: 91% versus 58% *p*:0.02
Nutting et al., 2011^4^	H&N	3D‐CRT versus IMRT	4y LC: ∼80% versus ∼70%
Heemsbergen et al., 2013^1^	Prostate high risk	2D versus 3D	LR: 11% versus 28% *p*:0.012; nLR: 9% versus 22% *p*:0.025
Witte et al., 2021^2^	Prostate	Higher versus standard peri‐prostatic dose	5y RFS: HR:0.57 (0.4–0.8), *p*: < 0.001
Gupta et al., 2020^5^	H&N	3D‐CRT versus IMRT	10y LRC: 79% versus 69%

Abbreviations: 3D‐CRT, three‐dimensional conformal radiotherapy; bRFS, biochemical relapse‐free survival; H&N, head and neck; HR, hazard ratio; IMRT, intensity modulated radiotherapy; LC, local control; LR, local relapse; LRC, local‐regional control; nLR, non‐local relapse; RFS, relapse free survival.

Moreover, research comparing the outcomes of high‐risk prostate cancer patients treated with different field sizes indicated a higher rate of clinical failures in those treated with conformal fields, which deliver a lower dose to extra‐prostatic areas, underscoring the potential detriment of margin reduction.^1^ These findings highlight the complexities and potential risks associated with highly conformed RT techniques and treatments employing reduced margins.

The adoption of modern RT techniques offers significant benefits in reducing toxicity; however, it necessitates a balanced approach to treatment planning. Ensuring adequate tumor coverage remains a fundamental oncological principle that should not be compromised by the drive to minimize treatment margins. Each case must undergo a careful evaluation of treatment margins to ensure that technological advancements are leveraged judiciously, prioritizing patient safety and treatment efficacy. Adherence to standard contouring guidelines and definition of irradiation margins based on the analysis of available set‐up and organ motion verification systems, against defining target volumes from own perception, could be a rational option.

In conclusion, while the advancements in RT technology represent a leap forward in oncological care, they also impose a responsibility to apply these innovations thoughtfully. It is paramount to maintain a balance between minimizing harm to healthy tissues and achieving effective tumor control. As we embrace these technological strides, our enthusiasm must be tempered with caution, ensuring that our pursuit of precision does not inadvertently lead to compromised treatment outcomes.

In this context, it becomes crucial, especially in educational initiatives aimed at less‐resourced settings, to emphasize not just the adoption of more advanced RT techniques, but also the importance of appropriate management of setup errors and organ motion. Such an approach ensures that the focus remains not solely on advanced delivery methods but also on the fundamental aspects of treatment accuracy, safety, and outcome assessment. This holistic perspective is essential for ensuring that advancements in RT are leveraged to their fullest potential, enhancing patient care without sacrificing the principles of effective tumor control and patient safety. As practitioners, navigating these advancements with an informed and cautious approach will be key to optimizing outcomes and upholding the integrity of oncological treatment in varied resource settings.

*Respectfully*,
